# A Randomized Trial of Split Dose 3 L Polyethylene Glycol Lavage Solution, 2 L Polyethylene Glycol Lavage Combined With Castor Oil, and 1 L of Polyethylene Glycol Lavage Solution Combined With Castor Oil and Ascorbic Acid for Preparation for Colonoscopy

**DOI:** 10.3389/fmed.2019.00158

**Published:** 2019-07-05

**Authors:** Xu Tian, Bing Shi, Xiao-Ling Liu, Hui Chen, Wei-Qing Chen

**Affiliations:** ^1^Key Laboratory for Biorheological Science and Technology, Department of Gastroenterology, Chongqing University Cancer Hospital, Chongqing Cancer Institute, Chongqing Cancer Hospital, Chongqing University, Ministry of Education, Chongqing, China; ^2^Chongqing Key Laboratory of Translational Research for Cancer Metastasis and Individualized Treatment, Department of Gastroenterology, Chongqing University Cancer Hospital, Chongqing Cancer Institute, Chongqing Cancer Hospital, Chongqing, China

**Keywords:** castor oil, ascorbic acid, bowel preparation, polyethylene glycol, colonoscopy

## Abstract

**Background:** Castor oil (CaO) has the potential of halving the required volume of bowel preparation solution; however, no clinical trial investigated the efficacy of CaO on bowel preparation for colonoscopy in addition to polyethylene glycol (PEG).

**Objectives:** Our aim was to evaluate efficacy and safety of lower dose PEG together with 30 mL CaO alone or plus ascorbic acid (Asc) in bowel preparation before colonoscopy.

**Methods:** Two hundred and forty-six patients were allocated randomly to ingest 2 L PEG with 30 mL CaO, 1 L PEG with 30 mL CaO plus 5 g Asc, or 3 L PEG. We used Boston Bowel Preparation Scale (BBPS) to evaluate bowel preparation efficacy. We also determined other outcomes such as procedure time, polyp or adenoma detection rate, and adverse events (AEs).

**Results:** Of 282 patients recruited, 36 were excluded. Groups were matched for baseline characteristics except weight (*P* = 0.020) and body mass index (BMI) (*P* = 0.003). Patient's satisfaction was higher in 2 L PEG-CaO (*P* = 0.016) and 1 L PEG-CaO-Asc groups (*P* = 0·017). Patients' compliance was 67.5, 71.4, and 80.5% in 3 L PEG, 2 L PEG-CaO, and 1 L PEG-CaO-Asc groups (*P* = 0.014). Adequate bowel preparation rate was 75, 78.57, and 53.66% in 3 L PEG, 2 L PEG-CaO, and 1 L PEG-CaO-Asc groups (*P* = 0.021). There were no differences in terms of remaining outcomes.

**Conclusions:** Despite an increase in patients' satisfaction and compliance, 1 L PEG-CaO-Asc significantly decreased adequate bowel preparation rate. However, 2 L PEG-CaO improved the patients' satisfaction and compliance and increased adequate bowel preparation rate (Registration number, ChiCTR-IIR-17012418).

## Introduction

Colorectal cancer (CRC) remains the major contributor to cancer-related morbidity and mortality (1). Colonoscopy been considered to be the preferred tool for effectively screening and early treating CRC (2). Issued data showed an approximate 50% reduction in mortality of CRC after resection of abnormal colonic lesions were performed by colonoscopy (3, 4). However, poor quality of bowel preparation will significantly decrease the efficacy and safety of colonoscopy procedure (5). Published data suggested that inadequate bowel preparation was directly associated with more than 40% of colonoscopy failures (6). Moreover, inadequate bowel preparation was related to lower polyp or adenoma detection rate (7), longer operation time (8), and higher risk of procedure related complications and incomplete colonoscopy rate (9).

To date, polyethylene glycol (PEG) solutions remain the preferred option of bowel preparation before colonoscopy (10). However, required high volume of liquid obviously reduces patients' tolerability and compliance (11). Thus, adjunctive drugs such as bisacodyl and ascorbic acid (Asc) have been added into PEG solutions in order to minimize the required volume of liquid (10, 12). However, desired quality of bowel preparation has not already been achieved. Consequently, it remains an open question how to improve bowel preparation efficacy before colonoscopy.

Castor oil (CaO) was derived from the seed of Ricinus communishas and has been widely used as a safe and effective stimulant laxative for colon cleansing in many settings (13–16). For example, Apisarnthanarak et al. (13) detected comparative patients' satisfaction and efficacy of colon cleansing between CaO and sodium phosphate. Yang et al. (16) unfolded that the laxative efficacy of CaO was comparable with that of bisacodyl. It is noted that the regime of bisacodyl plus PEG (17) and the regime of sodium and phosphate (18) achieved desired quality of bowel preparation, decreased the required volume of liquid, and improved compliance with the recommended regime when compared to standard PEG regime. Moreover, study also suggested that 2 L PEG containing Asc obtained similar bowel preparation efficacy with 3 L PEG (19), and which was superior to that of 2 L PEG with sodium phosphate (NaP) (20). So, we rationally speculated that 2 L PEG containing CaO may have comparative efficacy with 2 L PEG with bisacodyl or NaP in colon cleansing, and which is not inferior to high-volume 3 L PEG regime. Moreover, two trials (21, 22) suggested that 1 L PEG with bisacodyl and Asc was associated with improved patient's tolerability and desired quality of bowel preparation compared with 2 L PEG with Asc. Consequently, we also speculated that CaO plus Asc may halve the required liquid of PEG solutions.

Previous studies (23–25) suggested a higher rate of adverse effects such as abdominal cramping, abdominal fullness, nausea, vomiting, fainting, and insomnia after orally taken a large dose 50 or 60 mL of CaO. However, some trials found that low dose 30 mL CaO did not obviously increase the incidence of adverse events (13, 26, 27). Thus, we hypothesized that 30 mL CaO may enhance colon cleansing of PEG, and 30 mL CaO plus Asc (28) may halve the required liquid volume of PEG. The aim of the present trial was to ascertain the efficacy and safety of 3 L PEG lavage solution, 2 L PEG lavage combined with CaO, and 1 L PEG lavage solution combined with CaO and Asc for preparation for colonoscopy.

## Materials and Methods

### Study Design

A single-center, randomized, observer-blinded three-arm study was conducted from October 2017 to December 2018 at the endoscopy center at Chongqing University Cancer Hospital (Chongqing, China). In total, 82 patients received lower volume 1 L PEG with CaO plus Asc (1 L-PEG-CaO-Asc), 84 received low volume 2 L PEG with CaO (2 L-PEG-CaO), or 80 patients received traditional volume 3 L PEG. At the time of registration, subjects were randomly assigned to one of three groups. They were randomized by a computer-generated list and were provided with written instructions. All patients provided written informed consent before taking part in the present study. We obtained ethical approval from the Ethics Committee of Chongqing University Cancer Hospital and Chongqing Cancer Hospital. The trial is registered at Chinese Clinical Trial Registry (www.chictr.org.cn) with identifier ChiCTR-IIR-17012418.

### Patients

#### Inclusion Criteria

Participants met the following criteria were considered: (1) age above 18 and under 75 years; (2) adult outpatients who will be scheduled to morning colonoscopy regardless of sex; (3) did not participate in other clinical trials which also aimed at investigating bowel preparation efficacy; (4) agree to participate, and give signed written informed consents.

#### Exclusion Criteria

We excluded patients who met following criteria: (1) lactation; (2) pregnancy; (3) experienced the abdominal surgery such as gynecologic surgery, appendectomy, and laparoscopy; (4) neurological diseases; (5) contraindication of colonoscopy, (6) allergy to ingredients of PEG, CaO, or Asc or (6) other reasons such as uncontrolled severe hypertension and electrolyte imbalance that are considered to be unsuitable for study participation by the responsible investigators.

### Recruitment, Randomization, and Blind

The direct investigators who have been certified for colonoscopy examination by Medical Committee of Chongqing University Cancer Hospital and have completed 3,000 examinations in colonoscopy assessed the eligibility of each candidate according to the inclusion and exclusion criteria the day before colonoscopy. After identified the eligibility, direct investigators instructed patients, their next of kin or legal representatives to complete the written informed consents.

Research team generated the random sequence using SPSS 22.0 software, and the random sequence was sealed in opaque envelope. Then, an independent research nurse to randomly divided recruited patients into one of the three groups as following on the basis of a table of random numbers: 3 L PEG group, 2 L-PEG-CaO group, and 1 L-PEG-CaO-Asc group. The day of colonoscopy, the research nurse case report form to collect demographic and clinical characteristics data of all eligible patients, which includes sex, age, body weight, body mass index, indications for colonoscopy, previous colonoscopy history, and comorbidity such as hypertension, diabetes mellitus, and cardiovascular disease.

In order to eliminate the risk of bias as much as possible, the endoscopists were blinded except the research nurse who conducted the randomization procedure during examine period. Moreover, research team also blinded the biostatistician.

### Colonoscopy Preparation

According to the findings from our previous meta-analysis (29), all participants enrolled in our study were instructed to take low fat and residue diet without food color the 3 day before colonoscopy examination, and all started to fast at 20:00 pm on the day before colonoscopy examination. Patients were allowed eating bun, bread, and chocolate in order to enhance tolerance, decrease incidence of AEs such as hypoglycemia if they experienced serious hunger feeling. Moreover, investigators explained the purpose of colonoscopy and the importance of adequate bowel preparation before colonoscopy examination. In order to obtain adequate bowel preparation and take the fear away, investigators also explained the processes of bowel preparation for patients and the methods of processing all possible AEs associated with bowel preparation.

Moreover, the study protocols of all three groups have been outlined in our published protocol (30). According to the recommendation from US MultiSociety Task Force on Colorectal Cancer, we instructed all patients to ingest bowel preparation regime with split-dose. So, patients in 3 L PEG alone group were instructed to ingest 1 L PEG solution at 20:00 to 21:00 p.m. the day before colonoscopy and the remaining 2 L solution at the 3:00 to 5:00 a.m. on the morning before colonoscopy; patients in 2 L-PEG-CaO group were instructed to ingest 1 L PEG solution and 30 mL CaO at 20:00 to 21:00 p.m. the day prior to colonoscopy and the remaining 1 L PEG and extra 1 L clean water at the 3:00 to 5:00 a.m. on the morning before colonoscopy; patients in 1 L-PEG-CaO-Asc group were instructed to ingest 0.5 L PEG solution, 30 mL CaO, and 5 g Asc and extra 0.5 L clean water at 20:00 to 21:00 p.m. the day before colonoscopy and the remaining 0.5 L PEG solution and extra 1.5 L clean water at the 3:00 to 5:00 a.m. on the morning before colonoscopy. The patients were instructed to digest PEG solution 250 mL every 15 min. Moreover, all eligible participants were admitted to take oral 20 mL simethicone and 20 mL clean water at the 30 min prior to colonoscopy. For all eligible patients, propofol injection was intravenously administered for sedation.

### Study Endpoints

#### Primary Outcome

We defined the bowel preparation efficacy and adequate bowel preparation rate as the primary outcome in the present study (30). The direct operation doctor used the Boston Bowel Preparation Scale (BBPS) to evaluate the quality of bowel preparation (31). BBPS is a comprehensive scoring system of evaluating bowel preparation efficacy before colonoscopy (31), and has been widely used in clinical practice worldwide (32, 33). Details of BBPS have been described in our published study protocol (30). In the present study, the quality of bowel preparation was defined as adequate when bowel preparation achieved excellent or good for each segment.

#### Secondary Outcomes

We defined cecal intubation time, withdraw time, cecal intubation success rate, detection rate of polyp and adenoma, patients' satisfaction, tolerability and willingness to repeat colonoscopy, and quality of sleep as secondary outcomes. Cecal intubation time (endoscopists recorded the time of started colonoscopy examination until colonoscopy reached ileocecal part), withdraw time (endoscopists recorded the time of completely withdrew colonoscopy from anus), cecal intubation success rate (the proportion of successfully reached ileocecal part), and detection rate of polyp and adenoma (the proportion of polyp and adenoma detecte in the whole colonoscopy procedure) were measured by direct investigator. Patients' satisfaction (patients answered the questioner through selecting yes or no), tolerability [patients expressed feeling with a Likert scale ranged from 1 (not good) to 4 (excellent)] and willingness to repeat colonoscopy (patients expressed feeling to repeat colonoscopy through selecting yes or no), and quality of sleep (patients were instructed to self-evaluate the quality of sleep through answering the same or worse when it was compared to the previous night's sleep quality) were evaluated by research nurse with self-designed questionner.

#### Safety Assessments

The direct investigator recorded all AEs related to bowel preparation and colonoscopy such as abdominal fullness, abdominal pain, nausea, vomiting, and others into the case report form. It is noted that any symptom that existed before the start of the bowel preparation was not be recorded as AEs.

### Sample Size and Statistical Analysis

The bowel preparation efficacy was primarily tested in the present study, and thus we calculated the anticipated sample size based on this outcome. Based on the findings from previous studies (19, 21), we proposed that the rate of adequate bowel preparation in 3 L PEG, 2 L PEG with 30 mL CaO, and 1 L PEG with 30 mL CaO plus Asc will be 85, 90, and 95%. We assumed the significance and power to be 0.05 and 80%, respectively, and thus the sample size required to detect a difference will be 255 patients according to the non-inferiority design. Because the dropout rate was expected to be 10%, each trial group will be made up of at least 94 participants.

Continuous variables were expressed as mean ± standard deviation (SD), and discontinuous variables were expressed as counts and percentages. Data were analyzed on a Full Analysis Set basis with SPSS for Windows release 22.0 software (SPSS, Chicago, Illinois, USA). χ2 analysis or Fisher's exact test was used for comparison of categorical data. Normally distributed continuous data were analyzed by means of one-way ANOVA. Kruskal–Wallis *H*-test was used only for analysis of non-normally distributed data. Differences were considered significant at *P* < 0.050.

## Results

### Baseline Characteristics

**Figure 1:**
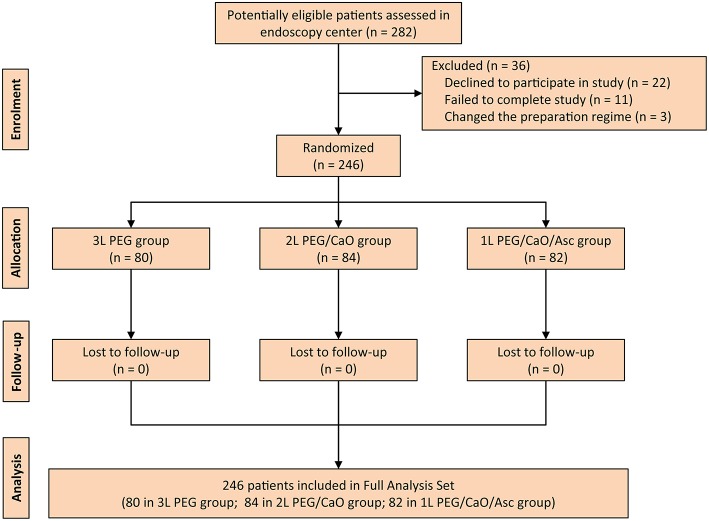
CONSORT diagram for the trial. PEG, polyethylene glycol; CaO, castor oil; Asc, ascorbic acid.

During the study period, a total of 282 consecutive patients were screened, but 36 patients were excluded due to various reasons including declined to participate in study (22 patients), failed to complete study (11 patients), and changed the preparation regime (three patients). Therefore, 246 patients were randomized and included in the full analysis set (FAS). A flow diagram that describes patients' enrollment is depicted in Figure 1.

The three groups were comparable in terms of age, sex, height, medical conditions, the indication for colonoscopy, previous colonoscopy, willingness to repeat colonoscopy and quality of sleep (Table 1). The weight (*p* = 0.020) and body mass index (BMI) (*P* = 0.003) in 3 L PEG and 1 L PEG-CaO-Asc groups were higher than that in 2 L PEG-CaO group. The most common reasons for colonoscopy were abdominal pain/distention/discomfort, changed in bowel habit, and post-polypectomy surveillance.

**Table 1:** Characteristics of the patients.

	**Group A (*n* = 80)**	**Group B (*B* = 84)**	**Group C (*n* = 82)**	***P*-value^a^**
Age (mean ± SD, years)	48.98 ± 12.47	52.26 ± 12.07	52.66 ± 8.79	0.273
Sex, *n* (%)				0.930
Male	42 (52.5)	44 (52.4)	40 (48.8)	
Female	38 (47.5)	40 (47.6)	42 (51.2)	
Height (mean ± SD, cm)	162.10 ± 8.53	161.62 ± 7.33	160.88 ± 7.15	0.771
Weight (mean ± SD, kg)	62.08 ± 11.23	58.39 ± 8.29	65.17 ± 12.72	0.020
BMI (mean ± SD, kg)	23.62 ± 3.92	22.31 ± 2.60	25.13 ± 4.34	0.003
Medical conditions, *n* (%)				
No	54 (67.5)	58 (69.0)	50 (61.0)	0.510
DM	2 (2.5)	4 (4.8)	6 (7.3)	0.378
Hypertension	14 (17.5)	10 (11.9)	12 (14.6)	0.598
Cardiac disease	2 (2.5)	2 (2.4)	2 (2.4)	1.000
Multiple	4 (5.0)	0 (0.0)	4 (4.8)	0.113
Others	4 (5.0)	10 (11.9)	8 (9.8)	0.287
Indication for colonoscopy, *n* (%)				
Diarrhea	4 (5.0)	6 (7.1)	2 (2.4)	0.378
Constipation	0 (0.0)	1 (0.0)	2 (2.4)	0.659
Abdominal pain/distention/discomfort	30 (37.5)	38 (45.2)	44 (53.7)	0.118
Change in bowel habit	12 (15.0)	10 (11.9)	8 (9.8)	0.592
Change in stool characteristics	4 (5.0)	3 (3.6)	0 (0.0)	0.117
GI bleeding	0 (0.0)	4 (4.8)	2 (2.4)	0.171
Surveillance	14 (17.5)	12 (14.3)	8 (9.8)	0.357
Physical examination	6 (7.5)	2 (2.4)	10 (12.2)	0.052
Others	10 (12.5)	8 (9.5)	6 (7.3)	0.537
Previous colonoscopy, *n* (%)	34 (42.5)	30 (35.7)	28 (34.1)	0.506
Satisfied with bowel preparation, *n* (%)				0.032
Very good/good	70 (87.5)	82 (97.6)	80 (97.6)	
General/not good	10 (12.5)	2 (2.4)	2 (2.4)	
Completion of bowel preparation, *n* (%)				0.014
No	6 (7.5)	2 (7.1)	0 (0.0)	
Yes	74 (92.5)	82 (92.9)	82 (100.0)	
Willingness to repeat colonoscopy, *n* (%)				0.159
No	26 (32.5)	24 (28.6)	16 (19.5)	
Yes	54 (67.5)	60 (71.4)	66 (80.5)	
Quality of sleep, *n* (%)				0.078
No change	26 (32.5)	30 (35.7)	40 (48.8)	
Worse	54 (67.5)	54 (64.3)	42 (51.2)	

*Group A, B, and C represents 3 L PEG, 2 L PEG with CaO 30 mL, and 1 L PEG with Cao 30 mL plus Asc 5 g, respectively. 3 L PEG, 3 L PEG; 2 L PEG with CaO 30 mL, 2 L PEG with CaO 30 mL; 1 L PEG with Cao 30 mL plus Asc 5 g, 1 L PEG with CaO 30 mL plus Asc 5 g. BMI, body mass index; SD, standard deviation; CaO, castor oil; Asc, ascorbic acid*.

a *Statistical significance between groups was tested by one-way ANOVA or Pearson χ^2^ analysis (Fisher's exact test if cell <5)*.

### Primary Outcome

**Figure 2:**
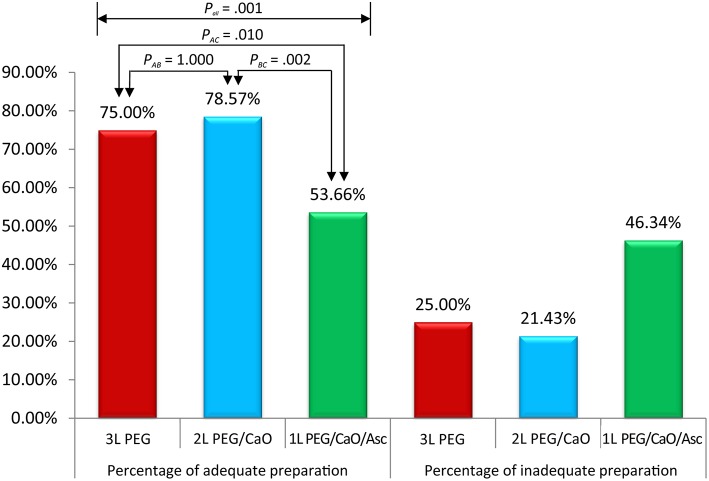
Percentages of adequate and inadequate bowel preparation among three groups. P_*all*_ corresponded to comparison of three groups, P_*AB*_ corresponded to comparison between 3 L PEG and 2 L PEG/CaO groups, P_*AC*_ corresponded to comparison between 3 L PEG and 1 L PEG/CaO/Asc groups, and P_*BC*_ corresponded to comparison between 2 L PEG/CaO and 1 L PEG/CaO/Asc groups. All comparisons were tested by one-way ANOVA or Pearson χ2 analysis (Fisher's exact test if cell <5). A, B, and C represents 3 L PEG, 2 L PEG/CaO, and 1 L PEG/CaO/Asc, respectively. PEG, polyethylene glycol; CaO, castor oil; Asc, ascorbic acid.

All methods showed no significant difference in terms of quality of bowel preparation scoring, with a mean (SD) total score of 6.95 ± 1.83 for the 3 L PEG group, 7.29 ± 1.60 for 2 L PEG-CaO-Asc group and 6.35 ± 1.83 for the 1 L PEG-CaO-Asc (*P* = 0.062). The analysis of the segmental (right, mid, and recto-sigmoid colon) BBPS scale showed no difference for the right side (2.26 ± 0.76 vs. 2.27 ± 0.71 vs. 1.97 ± 0.8), mid colon (2.26 ± 0.76 vs. 2.44 ± 0.59 vs. 2.08 ± 0.6), and recto-sigmoid colon (2.42 ± 0.60 vs. 2.59 ± 0.59 vs. 2.30 ± 0.74). Table 2 presents the results of bowel cleansing quality assessment based on the BBPS. Percent of adequate bowel preparation, defined as total BBPS score ≥6 was 75.0, 78.57, and 53.6% in 3 L PEG, 2 L PEG-CaO, and 1 L PEG-CaO-Asc groups, and the difference was statistically significant (*p* = 0.001) (Figure 2). Further analysis based on paired comparison found that 3 L PEG (*P* = 0.010) and 2 L PEG-CaO (*P* = 0.002) significantly increased the percent of adequate bowel preparation compared to 1 L PEG-CaO-Asc regime (Figure 2 and Table 3). Moreover, the reasons for incomplete colonoscopy mainly were extremely poor preparation and intolerance, however the percent of extremely poor preparation in 1 L PEG-CaO-Asc group was significantly higher than that in 3 L PEG (7.3 vs. 0.0%) and 2 L PEG-CaO (7.3 vs. 2.4%) groups.

**Table 2:** Efficacy of bowel cleansing assessed by Boston Bowel Preparation Scale.

	**Group A (*n* = 80)**	**Group B (*n* = 84)**	**Group C (*n* = 82)**	***P*-value^a^**
Right side of colon, (mean ± SD)	2.26 ± 0.76	2.27 ± 0.71	1.97 ± 0.87	0.172
Mid colon, (mean ± SD)	2.26 ± 0.76	2.44 ± 0.59	2.08 ± 0.68	0.072
Recto-sigmoid colon, (mean ± SD)	2.42 ± 0.60	2.59 ± 0.59	2.30 ± 0.74	0.145
Total score, (mean ± SD)	6.95 ± 1.83	7.29 ± 1.60	6.35 ± 1.83	0.062

*Group A, B, and C represents 3 L PEG, 2 L PEG with CaO 30 mL, and 1 L PEG with Cao 30 mL plus Asc 5 g, respectively. 3 L PEG, 3 L PEG; 2 L PEG with CaO 30 mL, 2 L PEG with CaO 30 mL; 1 L PEG with Cao 30 mL plus Asc 5 g, 1 L PEG with CaO 30 mL plus Asc 5 g. SD, standard deviation; CaO, castor oil; Asc, ascorbic acid*.

a *Statistical significance between groups was tested by one-way ANOVA*.

**Table 3:** Characteristics of the colonoscopy procedures.

	**Group A (*n* = 80)**	**Group B (*n* = 84)**	**Group C (*n* = 82)**	***P*-value^a^**
Cecal intubation success, n (%)	76 (95.0)	80 (95.2)	74 (90.2)	0.343
Reason for incomplete colonoscopy, n (%)				
Extremely poor preparation	0 (0.0)	2 (2.4)	6 (7.3)	0.021
Intolerance	2 (2.5)	2 (2.4)	1 (1.2)	0.871
Others	2 (2.5)	0 (0.0)	1 (1.2)	0.214
Adequate bowel preparation, n (%)	60 (75.0)	66 (78.57)	44 (53.66)	0.001
Cecal intubation time (min, mean ± SD)	7.84 ± 5.49	9.85 ± 12.75	10.19 ± 6.19	0.463
Withdrawal time (min, mean ± SD)	6.26 ± 2.36	6.20 ± 3.44	6.62 ± 4.81	0.864
Medical results, n (%)				
Normal	18 (22.5)	14 (16.7)	18 (22.0)	0.588
Polyps	8 (10.0)	20 (23.8)	16 (19.5)	0.063
Adenoma	26 (32.5)	25 (29.8)	24 (29.3)	0.891
Cancer	2 (2.5)	1 (1.0)	0 (0.0)	0.323
Colitis	22 (27.5)	22 (26.2)	24 (29.3)	0.906
Others	4 (5.0)	2 (2.4)	1 (1.2)	0.313

*Group A, B, and C represents 3 L PEG, 2 L PEG with CaO 30 mL, and 1 L PEG with Cao 30 mL plus Asc 5 g, respectively. 3 L PEG, 3 L PEG; 2 L PEG with CaO 30 mL, 2 L PEG with CaO 30 mL; 1 L PEG with Cao 30 mL plus Asc 5 g, 1 L PEG with CaO 30 mL plus Asc 5 g. SD, standard deviation; CaO, castor oil; Asc, ascorbic acid*.

a *Statistical significance between groups was tested by one-way ANOVA or Pearson χ^2^ analysis (Fisher's exact test if cell <5)*.

### Secondary Outcome

Details of colonoscopy procedures are summarized in Table 3, the cecal intubation rate of all groups was >90%, the insertion time was about 6 min, and the average withdrawal time was >7 min. The endoscopic diagnoses of the three groups were comparable, about 20% of the patients had no abnormal findings, over 20% of the patients were found to have colitis, over 40% of the patients were found to have colorectal polyps, in addition, more than 55% of the polyps were adenomas. Only very few patients had cancer (3/246, 1.2%). Patients in 2 L PEG-CaO and 1 L PEG-CaO-Asc groups were more satisfied with the process of bowel preparation than patients in 3 L PEG group (97.6 vs. 97.6 vs. 87.5%, *p* = 0.032). 92.5% and 92.9% patients in 3 L PEG and 2 L PEG-CaO groups completed bowel preparation, whereas all the patients in 1 L PEG-CaO-Asc group accomplished bowel preparation (*p* = 0.014). In addition, only 67.5% patients in 3 L PEG group were willing to repeat colonoscopy in the endoscopy center if necessary, but 71.4 and 80.5% patients in 2 L PEG-CaO and 1 L PEG-CaO-Asc groups were willing to do so although significant results were not detected (*P* = 0.159). There were no significant differences in quality of sleep (Table 1), the rates of abdominal fullness, abdominal pain, nausea, vomiting, and other AEs among three groups (Table 4).

**Table 4:** Characteristics of adverse events occurred in three groups.

	**Group A (*n* = 80)**	**Group B (*n* = 84)**	**Group C (*n* = 82)**	***P*-value^a^**
No adverse events (AEs), n (%)	14 (18.0)	16 (19.0)	20 (24.0)	0.518
Abdominal fullness, n (%)	6 (8.0)	4 (5.0)	10 (12.0)	0.209
Abdominal pain, n (%)	9 (11.0)	8 (10.0)	8 (10.0)	0.925
Nausea, n (%)	12 (15.0)	14 (17.0)	5 (6.0)	0.089
Vomiting, n (%)	6 (8.0)	4 (5.0)	5 (6.0)	0.711
Others, n (%)	0 (0.0)	1 (1.0)	1 (1.0)	1.000

*Group A, B, and C represents 3 L PEG, 2 L PEG with CaO 30 mL, and 1 L PEG with Cao 30 mL plus Asc 5 g, respectively. 3 L PEG, 3 L PEG; 2 L PEG with CaO 30 mL, 2 L PEG with CaO 30 mL; 1 L PEG with Cao 30 mL plus Asc 5 g, 1 L PEG with CaO 30 mL plus Asc 5 g. AEs, adverse event; SD, standard deviation; CaO, castor oil; Asc, ascorbic acid*.

a *Statistical significance between groups was tested by the Pearson χ^2^ analysis (Fisher's exact test if cell <5)*.

## Discussion

Although many novel and promising approaches have been proposed, colonoscopy remains a routine method of screening and early treating CRC (2). However, the quality of bowel preparation will significantly affect the efficacy and safety of colonoscopy examination (5), and evidence suggests about 25% of inadequate bowel preparation before colonoscopy (5). It must be important to note that poor bowel preparation will also increase the rate of incomplete colonoscopy and adverse events and lower polyp and adenoma detection rate (34, 35). Thus, several methods have been proposed to improve the quality of bowel preparation (36–38). Of these all methods, PEG solutions remain the first-line recommendation for bowel preparation prior to colonoscopy due to desire laxative efficacy (10), however required high volume of liquid will reduce tolerability and compliance to bowel preparation (11). Thus, numerous studies have been performed to explore the potential of reducing the volume of the cleansing solution by adding adjunctive prokinetics such as bisacodyl (39, 40), but the evidence suggests that gastrointestinal prokinetics can induce dose-dependent cardiac adverse effects (41). So, it is important to find a novel adjunctive laxative.

CaO is extracted from the seed of the castor-oil plant (42). CaO has a high content of the hydroxylated unsaturated fatty acid ricinoleic acid (43), and it has been demonstrated that released ricinoleic acid has the ability of inducing strong laxative effect by activating small-intestinal smooth-muscle cells via the EP3 prostanoid receptor (44). Moreover, CaO will not cause serious side effects (27), and thus it has been used as a safe stimulant laxative in many settings (14–16) except for pregnant women (45). Evidence suggested low dose CaO (30 mL) has similar laxative efficacy of cleaning colon to bisacodyl (16). Moreover, studies (26, 27) also showed that 30 mL of CaO can reduce volume of preparation of bowel preparation solutions. And thus, we designed a regime of 2 L split PEG with 30 mL CaO to perform bowel preparation before colonoscopy. The findings of our randomized controlled trial suggested that 2 L split PEG plus 30 mL CaO can increase the adequate bowel preparation rate and patients' satisfaction toward and patients' compliance with regime with comparable BBPS score and AEs rate compared to traditional 3 L split PEG solution.

A number of studies found that PEG with Asc regime obtained comparative efficacy, acceptability, tolerability, and safety related to the standard PEG regime (6, 11, 46, 47). Moreover, a recent meta-analysis also demonstrated the efficacy and safety of low-volume PEG containing Asc regime for bowel cleansing (48). Asc produces cathartic effects because of it will become saturated at a high dose (49, 50). Asc contribute toward decreasing the total volume of PEG solution required for gut lavage and improve patient's tolerability (27). For these reasons, we have further designed a lower-volume PEG preparation with 30 mL CaO plus 5 g Asc. The finding of our study showed that this modified lower-volume PEG regime obtained higher patients' satisfaction and compliance. However, it is noted that this modified bowel preparation regime significantly decreased the adequate bowel preparation rate compared with traditional 3 L split PEG regime. In the present study, patients with higher BMI were assigned to oral ingestion of 1 L split PEG with 30 mL CaO plus 5 g Asc. Studies have found that high BMI is an independent factor associated with inadequate bowel preparation for colonoscopy (51–53). This difference may be the contributor to the inconsistent finding. So, further studies are needed in order to determine the adjunctive efficacy of combination of CaO and Asc.

We must acknowledge that our study has some limitations. First, in this study, evaluation on electrolyte levels and hematological analysis were not performed either before or after colon preparation. However, no patient experienced significant adverse events related to bowel preparation and procedure. Second, we performed this study in single-center and obtained results supported by insufficient number of patients. Thus, we suggest to design a multi-center study with larger scale to perform a more precise assessment. Third, evaluation of electrolyte levels or hematological analysis was not carried out during the whole colonoscopy examination. However, we did not detect any significant difference in the rate of adverse events.

## Conclusions

In conclusion, the results of this study indicate that 30 mL CaO in addition to 2 L PEG before colonoscopy is safe, and it can improve patients' satisfaction toward and compliance with the process of bowel preparation, increase the adequate bowel preparation rate, and obtain equal quality of bowel preparation to 3 L split PEG. Both preparation methods were effective. Patient's adverse events and quality of sleep were similar between the two groups. However, patients taking 1 L PEG with 30 mL CaO plus Asc 5 mg in general showed more inadequate bowel preparation although it improved patients' satisfaction and compliance related to 3 L PEG regime.

## Data Availability

The raw data supporting the conclusions of this manuscript will be made available by the authors, without undue reservation, to any qualified researcher.

## Ethics Statement

All patients provided written informed consent before taking part in the present study. We obtained ethical approval from the Ethics Committee of Chongqing University Cancer Hospital and Chongqing Cancer Hospital.

## Author Contributions

XT and W-QC: conceptualization and resources. XT and X-LL: data curation. XT: formal analysis, software, and visualization. W-QC: funding acquisition, project administration, supervision, writing—review, and editing. XT, BS, and HC: investigation. XT and BS: methodology. XT, BS, X-LL, and W-QC: validation. XT and HC: writing—original draft.

### Conflict of Interest Statement

The authors declare that the research was conducted in the absence of any commercial or financial relationships that could be construed as a potential conflict of interest.

## References

[1] Torre LA Bray F Siegel RL Ferlay J Lortet-Tieulent J Jemal A. Global cancer statistics, 2012. CA Cancer J Clin. (2015) 65:87–108. 10.3322/caac.21262 25651787

[2] Tian X Chen WQ Huang JL He LY Liu BL Liu X. Effects of polyethylene glycol 2 L alone or with ascorbic acid compared with polyethylene glycol 4 L alone for bowel preparation before colonoscopy: protocol for a systematic review and network meta-analysis. BMJ Open. (2017) 7:e018217. 10.1136/bmjopen-2017-018217 29042393 PMC5652536

[3] Quintero E Castells A Bujanda L Cubiella J Salas D Lanas A. Colonoscopy versus fecal immunochemical testing in colorectal-cancer screening. N Engl J Med. (2012) 366:697–706. 10.1056/NEJMoa1108895 22356323

[4] Zauber AG Winawer SJ O'Brien MJ Lansdorp-Vogelaar I van Ballegooijen M Hankey BF. Colonoscopic polypectomy and long-term prevention of colorectal-cancer deaths. N Engl J Med. (2012) 366:687–96. 10.1056/NEJMoa1100370 22356322 PMC3322371

[5] Froehlich F Wietlisbach V Gonvers JJ Burnand B Vader JP. Impact of colonic cleansing on quality and diagnostic yield of colonoscopy: the European panel of appropriateness of gastrointestinal endoscopy European multicenter study. Gastrointest Endosc. (2005) 61:378–84. 10.1016/S0016-5107(04)02776-2 15758907

[6] Ponchon T Boustiere C Heresbach D Hagege H Tarrerias AL Halphen M. A low-volume polyethylene glycol plus ascorbate solution for bowel cleansing prior to colonoscopy: the NORMO randomised clinical trial. Dig Liver Dis. (2013) 45:820–6. 10.1016/j.dld.2013.04.009 23769755

[7] Lebwohl B Kastrinos F Glick M Rosenbaum AJ Wang T Neugut AI. The impact of suboptimal bowel preparation on adenoma miss rates and the factors associated with early repeat colonoscopy. Gastrointest Endosc. (2011) 73:1207–14. 10.1016/j.gie.2011.01.051 21481857 PMC3106145

[8] Harewood GC Sharma VK de Garmo P. Impact of colonoscopy preparation quality on detection of suspected colonic neoplasia. Gastrointest Endosc. (2003) 58:76–9. 10.1067/mge.2003.294 12838225

[9] Rex DK Imperiale TF Latinovich DR Bratcher LL. Impact of bowel preparation on efficiency and cost of colonoscopy. Am J Gastroenterol. (2002) 97:1696–700. 10.1111/j.1572-0241.2002.05827.x 12135020

[10] Clark RE Godfrey JD Choudhary A Ashraf I Matteson ML Bechtold ML. Low-volume polyethylene glycol and bisacodyl for bowel preparation prior to colonoscopy: a meta-analysis. Ann Gastroenterol. (2013) 26:319–24. 24714413 PMC3959486

[11] Choi HS Shim CS Kim GW Kim JS Lee SY Sung IK. Orange juice intake reduces patient discomfort and is effective for bowel cleansing with polyethylene glycol during bowel preparation. Dis Colon Rectum. (2014) 57:1220–7. 10.1097/DCR.0000000000000195 25203380

[12] Valiante F Pontone S Hassan C Bellumat A De Bona M Zullo A. A randomized controlled trial evaluating a new 2-L PEG solution plus ascorbic acid vs. 4-L PEG for bowel cleansing prior to colonoscopy. Dig Liver Dis. (2012) 44:224–7. 10.1016/j.dld.2011.10.007 22119219

[13] Apisarnthanarak P Rotjanaaree B Komoltri C Charoensak A Apisarnthanarak A Hargrove NS. Prospective, randomized comparison of castor oil and sodium phosphate preparation for barium enema. J Med Assoc Thai. (2009) 92:243–9. 19253801

[14] Bradley AJ Taylor PM. Does bowel preparation improve the quality of intravenous urography? Br J Radiol. (1996) 69:906–9. 10.1259/0007-1285-69-826-906 9038524

[15] Jansson M Geijer H Andersson T Bowel preparation for excretory urography is not necessary: a randomized trial. Br J Radiol. (2007) 80:617–24. 10.1259/bjr/78311002 17681986

[16] Yang HC Sheu MH Wang JH Chang CY. Bowel preparation of outpatients for intravenous urography: efficacy of castor oil versus bisacodyl. Kaohsiung J Med Sci. (2005) 21:153–8. 10.1016/S1607-551X(09)70294-3 15909670 PMC11917964

[17] Brahmania M Ou G Bressler B Ko HK Lam E Telford J. 2 L versus 4 L of PEG3350 + electrolytes for outpatient colonic preparation: a randomized, controlled trial. Gastrointest Endosc. (2014) 79:408–16.e4. 10.1016/j.gie.2013.08.035 24206747

[18] Bae SE Kim KJ Eum JB Yang DH Ye BD Byeon JS. A Comparison of 2 L of polyethylene glycol and 45 mL of sodium phosphate versus 4 L of polyethylene glycol for bowel cleansing: a prospective randomized trial. Gut Liver. (2013) 7:423–9. 10.5009/gnl.2013.7.4.423 23898382 PMC3724030

[19] Zhang S Li M Zhao Y Lv T Shu Q Zhi F. 3-L split-dose is superior to 2-L polyethylene glycol in bowel cleansing in Chinese population: a multicenter randomized, controlled trial. Medicine. (2015) 94:e472. 10.1097/MD.0000000000000472 25634195 PMC4602972

[20] Cohen SM Wexner SD Binderow SR Nogueras JJ Daniel N Ehrenpreis ED. Prospective, randomized, endoscopic-blinded trial comparing precolonoscopy bowel cleansing methods. Dis Colon Rectum. (1994) 37:689–96. 10.1007/BF02054413 8026236

[21] Ji EK Lee JW Im JP Ji WK Su HK Koh SJ Comparable efficacy of a 1-L PEG and ascorbic acid solution administered with bisacodyl versus a 2-L PEG and ascorbic acid solution for colonoscopy preparation: a prospective, randomized and investigator-blinded trial. PLoS ONE. (2016) 11: e0162051 10.1371/journal.pone.0162051 27588943 PMC5010253

[22] Kang SH Lee JH Yoo IK Lee JM Kim SH Choi HS Sa1472 A comparison of bowel preparation between 2L ascorbic acid mixed PEG and 1L ascorbic acid mixed PEG with bisacodyl. Gastrointest Endosc. (2016) 83:AB287 10.1016/j.gie.2016.03.448

[23] Avgerinos A Kalantzis N Rekoumis G Pallikaris G Arapakis G Kanaghinis T. Bowel preparation and the risk of explosion during colonoscopic polypectomy. Gut. (1984) 25:361. 10.1136/gut.25.4.361 6423457 PMC1432339

[24] Chen CC Ng WW Chang FY Lee SD. Magnesium citrate-bisacodyl regimen proves better than castor oil for colonoscopic preparation. J Gastroenterol Hepatol. (1999) 14:1219–22. 10.1046/j.1440-1746.1999.02032.x 10634161

[25] Ghazikhanlou SK Jafari MR Shams S A comparison of the efficacy, adverse effects, and patient compliance of the sena-graph®syrup and castor oil regimens for bowel preparation. Iran J Pharm Res. (2010) 9:193–8. 24363727 PMC3862068

[26] Hotta N The use of castor oil for bowel preparation for colon capsule endoscopy. Open J Med Imaging. (2016) 06:103–7. 10.4236/ojmi.2016.64010

[27] Ohmiya N Hotta N Mitsufuji S Nakamura M Omori T Maeda K. Multicenter feasibility study of bowel preparation with castor oil for colon capsule endoscopy. Dig Endosc. (2018) 31:164–72. 10.1111/den.13259 30102791

[28] Rivas JM Perez A Hernandez M Schneider A Castro FJ. Efficacy of morning-only 4 liter sulfa free polyethylene glycol vs 2 liter polyethylene glycol with ascorbic acid for afternoon colonoscopy. World J Gastroenterol. (2014) 20:10620–7. 10.3748/wjg.v20.i30.10620 25132784 PMC4130875

[29] Song GM Tian X Ma L Yi LJ Shuai T Zeng Z Regime for bowel preparation in patients scheduled to colonoscopy: low-residue diet or clear liquid diet? evidence from systematic review with power analysis. Medicine. (2016) 95:e2432 10.1097/MD.0000000000002432 26735547 PMC4706267

[30] Tian X Chen WQ Liu XL Chen H Liu BL Pi YP. Comparative efficacy of combination of 1 L polyethylene glycol, castor oil and ascorbic acid versus 2 L polyethylene glycol plus castor oil versus 3 L polyethylene glycol for colon cleansing before colonoscopy. Medicine. (2018) 97:e0481. 10.1097/MD.0000000000010481 29703007 PMC5944546

[31] Heron V Parmar R Menard C Martel M Barkun AN. Validating bowel preparation scales. Endosc Int Open. (2017) 5:E1179–88. 10.1055/s-0043-119749 29202001 PMC5698009

[32] Calderwood AH Jacobson BC. Comprehensive validation of the Boston Bowel Preparation Scale. Gastrointest Endosc. (2010) 72:686–92. 10.1016/j.gie.2010.06.068 20883845 PMC2951305

[33] Gao Y Lin JS Zhang HD Lin MX Cheng CS Wu SZ. Pilot validation of the Boston bowel preparation scale in China. Dig Endosc. (2013) 25:167–73. 10.1111/j.1443-1661.2012.01356.x 23368700

[34] Chokshi RV Hovis CE Hollander T Early DS Wang JS. Prevalence of missed adenomas in patients with inadequate bowel preparation on screening colonoscopy. Gastrointest Endosc. (2012) 75:1197–203. 10.1016/j.gie.2012.01.005 22381531

[35] Clark BT Tarun R Loren L. What level of bowel prep quality requires early repeat colonoscopy: systematic review and meta-analysis of the impact of preparation quality on adenoma detection rate. Am J Gastroenterol. (2014) 109:1714–23. 10.1038/ajg.2014.232 25135006 PMC4423726

[36] Chang CW Shih SC Wang HY Chu CH Wang TE Hung CY. Meta-analysis: the effect of patient education on bowel preparation for colonoscopy. Endosc Int Open. (2015) 3:E646–52. 10.1055/s-0034-1392365 26716129 PMC4683152

[37] Lichtenstein GR Cohen LB Uribarri J. Review article: Bowel preparation for colonoscopy–the importance of adequate hydration. Aliment Pharmacol Ther. (2010) 26:633–41. 10.1111/j.1365-2036.2007.03406.x 17697197

[38] Myriam M Barkun AN Charles M Sophie R Omar K Alain V Split-dose preparations are superior to day-before bowel cleansing regimens: a meta-analysis. Gastroenterology. (2015) 149:79–88. 10.1053/j.gastro.2015.04.004 25863216

[39] Martínek J Hess J Delarive J Jornod P Blum A Pantoflickova D Cisapride does not improve precolonoscopy bowel preparation with either sodium phosphate or polyethylene glycol electrolyte lavage. Gastrointest Endosc. (2001) 54:180–5. 10.1067/mge.2001.116562 11474387

[40] Tajika M Niwa Y Bhatia V Kawai H Kondo S Sawaki A. Efficacy of mosapride citrate with polyethylene glycol solution for colonoscopy preparation. World J Gastroenterol. (2012) 18:2517–25. 10.3748/wjg.v18.i20.2517 22654449 PMC3360450

[41] Tonini M De Ponti F Di Nucci A Crema F. Review article: cardiac adverse effects of gastrointestinal prokinetics. Aliment Pharmacol Ther. (1999) 13:1585–91. 10.1046/j.1365-2036.1999.00655.x 10594392

[42] Gaginella TS Phillips SF. Ricinoleic acid: current view of an ancient oil. Am J Dig Dis. (1975) 20:1171–7. 10.1007/BF01070759 1200010

[43] Watson WC Gordon RS Jr. Studies on the digestion, absorption and metabolism of castor oil. Biochem Pharmacol. (1962) 11:229–36. 10.1016/0006-2952(62)90078-3 14005307

[44] Tunaru S Althoff TF Nüsing RM Martin D Stefan O. Castor oil induces laxation and uterus contraction via ricinoleic acid activating prostaglandin EP3 receptors. Proc Natl Acad Sci USA. (2012) 109:9179–84. 10.1073/pnas.1201627109 22615395 PMC3384204

[45] Sicuranza GB Figueroa R. Uterine rupture associated with castor oil ingestion. J Matern Fetal Neonatal Med. (2003) 13:133–4. 10.1080/713605796 12735415

[46] Marmo R Rotondano GG Marone A Bianco MA Stroppa I Caruso A. Effective bowel cleansing before colonoscopy: a randomized study of split-dosage versus non-split dosage regimens of high-volume versus low-volume polyethylene glycol solutions. Gastrointest Endos. (2010) 72:313–20. 10.1016/j.gie.2010.02.048 20561621

[47] Corporaal S Kleibeuker JH Koornstra JJ. Low-volume PEG plus ascorbic acid versus high-volume PEG as bowel preparation for colonoscopy. Scand J Gastroenterol. (2010) 45:1380–6. 10.3109/00365521003734158 20602568

[48] Xie Q Chen L Zhao F Zhou X Huang P Zhang L. A meta-analysis of randomized controlled trials of low-volume polyethylene glycol plus ascorbic acid versus standard-volume polyethylene glycol solution as bowel preparations for colonoscopy. PLoS ONE. (2014) 9:e99092. 10.1371/journal.pone.0099092 24902028 PMC4047058

[49] Flavio Valiante Angelo Bellumat Manuela Bona Bisacodyl plus split 2-L P-citrate-simethicone improves quality of bowel preparation before screening colonoscopy. World J Gastroenterol. (2013) 19:5493–9. 10.3748/wjg.v19.i33.5493 24023492 PMC3761102

[50] Tajika M Tanaka T Ishihara M Mizuno N Hara K Hijioka S. A randomized controlled trial evaluating a low-volume peg solution plus ascorbic acid versus standard PEG solution in bowel preparation for colonoscopy. Gastroenterol Res Pract. (2015) 2015:1–12. 10.1155/2015/326581 26649036 PMC4662975

[51] Fayad NF Kahi CJ Abd El-Jawad KH Shin AS Shah S Lane KA. Association between body mass index and quality of split bowel preparation. Clin Gastroenterol Hepatol. (2013) 11:1478–85. 10.1016/j.cgh.2013.05.037 23811246 PMC3805775

[52] Hyun JH Kim SJ Park JH Wie GA Kim JS Han KS. Lifestyle factors and bowel preparation for screening colonoscopy. Ann Coloproctol. (2018) 34:197–205. 10.3393/ac.2018.03.13 30208683 PMC6140368

[53] Sharara AI Harb AH Sarkis FS Chalhoub JM Habib RH. Body mass index and quality of bowel preparation: real life vs. clinical trials. Arab J Gastroenterol. (2016) 17:11–6. 10.1016/j.ajg.2015.12.001 26795085

